# Empirical findings of fitness-for-duty evaluations

**DOI:** 10.15694/mep.2018.0000258.1

**Published:** 2018-11-14

**Authors:** James Reich, Michael Kelly

**Affiliations:** 1 UCSF Medical School; 2 Stanford Medical School

**Keywords:** physician, impairment, fitness to practice, fitness for duty, psychiatry, psychiatric evaluation

## Abstract

This article was migrated. The article was marked as recommended.

**Background**:The ability of physicians to practice appropriately is often evaluated by a fitness for duty exam. This report reviews the empirical literature on fitness for duty evaluations.

**Methods**: A literature review was performed on PubMed using the terms physician, impairment, burnout, fitness to practice and fitness for duty.

**Results:** At least one percent of physicians are referred each year for possibly serious difficulties. Surgery and its subspecialties and psychiatry may be at higher risk. Variables associated with fitness for duty evaluations include educational, personality, culture and emotional illness.

**Conclusions**: Risk factors appear to vary between modifiable (training, culture and treatable emotional illness), less modifiable (personality) and likely unmodifiable (specialty).  Fitness for duty should be part of the training of all psychiatrists.

## Introduction

Medicine is a self-regulating profession in that it attempts to ensure that its practitioners are practicing within accepted standards of care. The question of whether a given decision or situation is within the standard of care is usually determined by specialists in that profession (e.g. surgeons assess surgeons, internists assess internists, etc.) However, when broader issues of functioning are to be assessed, a psychiatrist may be required to perform a fitness-for-duty evaluation. This report examined the empirical literature on this subject relevant to physicians.

## Methods

A literature review was performed on PubMed using the terms physician, impairment, burnout, fitness to practice and fitness for duty. References from articles identified by PubMed were also reviewed to find other relevant articles. Potential articles selected for this review were those that focused on fitness for duty, were empirically based, had a well described sample and a sample size greater than five. The findings were summarized and then applied to the educational vignettes given below.

### Vignettes

#1

Dr. X was a psychiatrist who successfully finished his residency training but never took his boards. After residency, he worked for a few community mental health centers before going into private practice. His private practice, largely focused on the medication treatment of psychiatric patients. Dr. X had a stable marriage as well as a close relationship with a fellow psychiatrist who lived a short distance away. In the evenings after work, he would stop at his friend’s house, where they would have a drink and discuss medical cases and non-work issues.

Things went well for Dr. X for a number of years, until his friend died. After his friend’s death, a number of complaints were made regarding Dr. X; his practice was described as being “a bit off,” although no urgent patient issues were reported. Problems related to billing were also reported, such as his liberal interpretation of medical billing codes for re-imbursement.

The medical board assigned Dr. X a supervising physician. Observation took place during Dr. X’s work hours. The supervisory physician noted that Dr. X would occasionally discuss a patient’s issues with the patient while the two were within earshot of other patients. Although the recent problems with billing and the discussion of sensitive health information in public settings were of concern, the supervising physician believed these issues were correctable.

A meeting was held with the medical board, Dr. X, and the supervisor. During a discussion of patient privacy, Dr. X became defensive, stating that he believed that all patients should be able to hear the details of all the other patients’ issues. The supervisor pointed out to the board (and Dr. X) that this was not what he had observed; rather, the supervisor reported, Dr. X did attempt to maintain privacy, but his attempts were at times unsuccessful. Dr. X then admitted that the supervisor’s observation was correct.

The board had Dr. X continue in supervised practice until the deficiencies in his ability to maintain patient privacy were corrected. Eventually Dr. X was allowed to practice without supervision.

#2

Dr. Y was a married, board-certified general surgeon who did well in medical school and residency and was respected by colleagues in his private practice. His wife of many years helped organize the couple’s social life. At some point the marriage began to deteriorate. As a result, the couple’s social life also declined. This was more of a problem for Dr. Y than his wife, who had her own friends to socialize with. Dr. Y began to spend more time at the office, where he felt appreciated by his colleagues and patients. Unfortunately, Dr. Y’s increased time at work further isolated him socially, and he began to drink more alcohol in the evenings. Dr. Y used the drinking as a means of helping him deal with the frequent arguments he had with his wife after returning home from work.

After one particularly nasty fight, Dr. Y told his wife he would sleep at a friend’s vacant apartment. Dr. Y began driving to his office and soon realized he had forgotten to bring along some food and other items. Rather than return home, Dr. Y drove to a supermarket. After selecting the items needed, Dr. Y realized he had forgotten his wallet at home and had only a few dollars in his pocket. Dr. Y was reluctant to return home for his wallet, decided to put some of the items in his pockets, and was subsequently arrested for theft. The incident was reported to the medical board after the arresting officer realized Dr. Y was a physician. Dr. Y’s license was placed on probation during the medical board’s investigation. During this time, Dr. Y engaged in psychotherapy and medication management for major depression and marital issues. Alcohol education was also provided. Dr. Y ultimately divorced, came to terms with his emotional issues, and was removed from probation. There were no patient complaints prior to or throughout the medical board’s involvement with Dr. Y.

## Results

*Scope of the problem.* Questions about a physician’s ability to practice are common and are of concern to both the public and the physician involved. In the United States (US) in 2015, the Federation of State Medical Boards (FSMB) took 7,942 actions (2016). Over 1,200 of these actions involved the restriction of physician licenses. Even more accurate tallies are available from the United Kingdom (UK)’s National Health Service. In 1994, Donaldson found that, over a five-year period, 6% of senior physicians working in the northern health region of the National Health Service were referred for questions of fitness-for-duty ( Donaldson, 1994). In 2009, the General Medical Council of the United Kingdom found that approximately 2.5% of physicians were referred for a question of fitness-to-practice (2010). Around half of these referrals were determined to be minor or insignificant; however, the remainder required some level of investigation. The frequency of disciplinary actions in the US, and data from the UK, suggests that questions surrounding the fitness-to-practice medicine are a frequent occurrence.

*Findings from studies of medical students.* Performance in medical school may be an early indicator of future problems related to fitness-for-duty. A small minority of medical students run into disciplinary problems in medical school. Braatvedt, ( Braatvedt et al., 2014) found that only 5.5% of medical students received fitness-for-duty evaluations while Howe et al. reported 3% ( 2010). It is important to note that, with counseling, virtually all of these students managed to return to good standing.

Howe’s research provides the most comprehensive summary of specific deficiencies leading to fitness-for-duty referrals in the medical student population ( Howe et al., 2010). Regarding reasons for referral, Howe reported unexplained absence at 26%; tutor reports unsatisfactory behavior at 21%; lack of meeting responsibilities at 16.4%; plagiarism at 12.3%; other behavioral at 12.3%; and falsification of signatures at 4.1%. Of note, Howe’s list includes infractions covering a wide range of behaviors, and there do not appear to be specific behaviors relative to a fitness-for-duty referral. Rather, unprofessional behavior in general appears to be the common link among fitness-for-duty referrals.

Regarding the association of demographic and personal characteristics with fitness-for-duty referrals during medical school, Braatvedt and colleagues found that referrals most often involved males in their clinical years ( Braatvdt et al., 2014). Papadakis et al. found that disciplinary action was significantly associated with prior unprofessional behavior in medical school (OR = 3.0) for a population-attributable risk of 26% ( Papdakis et al., 2005). The types of unprofessional behavior most strongly linked with disciplinary action were severe irresponsibility (OR = 8.5) and severely diminished capacity for self-improvement (OR = 3.0). Low MCAT scores and poor grades during the first two years of medical school were also associated with discipline but had very low attributable risk. An examination of five medical students, who each received two fitness-for-duty referrals, revealed that a poor relationship with the health care team was the most prevalent reason for referral ( Papadakis et al., 1999). In their examination of medical student personality factors, Dowell and colleagues found that students who did not rate on the extreme ends of personality measures tended to higher scores on measures of professionalism ( Dowell et al., 2011). It is worth noting, however, that the study by Dowell and colleagues had some limitations that included a high dropout rate and the use of study instruments with questionable validity. These data, and those obtained by Papadakis et al., indicate that deficiencies in professionalism factors may play a significant role in early medical school difficulties ( Papadakis et al., 2005).

Medical students referred for fitness-for-duty evaluations almost invariably return to the regular education process with counseling. However, there is evidence suggesting that a history of referral for a fitness-for-duty evaluation during medical school serves as a harbinger of future problems. For example, in the case control study performed by Papadakis et al., problematic behavior during medical school was associated with subsequent medical board discipline ( Papdakis et al., 2004). Most notably, a logistic regression (p = .02) showed that the variable of “Concern/Problem/Extreme” excerpts in one’s medical school file was the only significant predictor of medical board discipline. Teherani et al. carried this analysis further in an attempt to identify specific personality factors that associated with later medical board discipline. Three domains of unprofessional behavior significantly related to later disciplinary problems were identified: 1) poor reliability and responsibility; 2) lack of self-improvement and adaptability; and 3) poor initiative and motivation ( Teherani et al., 2005). Thus, the literature indicates that personality factors can play a role in professional difficulties during medical school and beyond.

**Table 1.** Empirical results of studies examining medical students who have possible impairment in their roles as physicians.

Study	Design	Number of subjects	Type of subjects	Variables measured	Outcome	Specialty
Braatvedt et al., 2014	Recording of results of fitness-for-duty reports for the years 2005-2013.	2,382	Medical students.	Fitness-for-duty reports on students.	132 students (5.5%) received reports. These were more often males and more often in clinical years. Ninety-six percent successfully completed the program after counselling.	
Dowell et al., 2011	Retrospective study of applicants to Scottish medical schools 2002-2003.	626	Medical students.	The Personal Quality Assessment (PQA) to examine non-cognitive factors. Objective structured clinical examination(OSCE) rankings were available on a subsample.	This study was limited by high attrition. There was one finding of significance: that those students who were identified by PQA as ‘not extreme’ on the two personalcharacteristics scales performed better in an OSCE measure of professionalism.	
Howe et al., 2010	Retrospective study on United Kingdom medical students.	5,867	Medical students.	Reported incidents possibly reflecting failure to be fit for duty	Only 3% of students (176) had a report although with some had more than one so the total level of reports was 195. The largest issues were: 1) unexplained absence 26%; 2) tutor reports unsatisfactory behavior 21%; 3) lack of meeting responsibilities 16.4%; 4) plagiarism 12.3%; 5) other behavioral 12.3%; and 6) falsification of signatures 4.1%.	
Papadakis et al., 1999	Descriptive study of medical students.	24	Medical students at UCSF Medical Center 1995-1998.	Citations for unprofessional behavior.	Of the five students that received two reports, the most common finding was a poor relationship with the health care team.	
Papadakis et al., 2005	Case control study.	235 cases;469 controls	Graduates of three medical schools.	Discipline by one of 40 state medical boards between 1990 and 2003.	Disciplinary action was significantly associated with prior unprofessionalbehavior in medical school (OR = 3.0) for a population-attributable risk of 26%.The types of unprofessional behavior most strongly linked with disciplinary action were severe irresponsibility (OR = 8.5) and severely diminished capacity for self-improvement (OR = 3.0). Low MCAT scores and poor grades the first two years of medical school were associated with discipline but with very low attributable risk.	
Teherani et. al.,2005	Retrospective case study.	68 cases;196 matched controls	Medical students.	Subsequent discipline by medical boards.	Three types of behavior were associated with later disciplinary outcome:1) poor reliability and responsibility; 2) lack of self-improvement and adaptability; and 3) poor initiative andmotivation.	
Papadakis et al., 2004	Case control.	68 cases; 196 matched controls	Medical students.	Subsequent discipline by a medical board.	Problematic behavior in medical school associated with subsequent medical board discipline. Ninety-five percent of the disciplinary actions were for deficiencies in professionalism.	

*Findings from the empirical literature on physician fitness-for-duty.* It is reasonable to ask: Do some specialties tend toward more fitness-for-duty difficulties? Donaldson examined a group of 49 out of 850 UK physicians who were considered “problem doctors” and found the following results (%): Anesthesiology: 14; General Medicine: 4: General Surgery: 8; Geriatrics: 8; Obstetrics and Gynecology: 10; Other Medical (including Pediatrics): 12; Other Surgical: 18; Psychiatry: 22; and Radiology: 2 ( Donaldson, 1994). Campbell’s research group provided specialty figures for a representative sample of UK physicians (%): Anesthesia: 1; General Practice: 2; Medicine: 2; Non-General Practice/Specialist: 1; Obstetrics and Gynecology: 4; Ophthalmology: 2; Pediatrics: 2; Pathology: 1; Psychiatry: 5; Radiology: 1; and Surgery: 4 ( Campbell, Hodgson Rollin and Smith, 2013). Finlayson and colleagues **e**xamined physicians referred to the Vanderbilt Assessment Service and found that the largest represented specialties were (%): Internal Medicine: 41.4; Surgery: 13.7; Family Medicine: 10.9; and Pediatrics: 8.7 ( Finlayson et al., 2013). Based on these data, it appears that physicians practicing in surgery and its subspecialties, and psychiatry, may be at higher risk for professional problems.

*Characteristics of those referred for evaluation.* Finlayson and colleagues documented the mental health diagnoses of those referred for fitness-for-duty evaluations at the Vanderbilt Assessment Service. Of those assessed (%), 36.7 had one Axis I diagnosis; 20.5 had two; 10.5 had three; and 1.8 had four ( Finalyson et al., 2013). These data show that a majority of those evaluated (69.5%) had a diagnosable emotional illness. Of note, the presence of an Axis I diagnosis reduced the chances of being found fit-for-duty. Physicians presenting with disruptive behavior were significantly less likely to be assessed as unfit for practice when compared to persons with a history of problematic substance use, mental health concerns, and/or poor sexual boundaries. Donaldson described the reasons for referral (%) as 1) Poor attitude and disruptive or irresponsible behavior: 65; 2) Lack of commitment to duties: 42; 3) Poor skills and inadequate knowledge: 39; 4) Dishonesty: 22; 5) Sexual matters: 14; 6) Disorganized practice and poor communication with colleagues: 10; and 7) Other problems: 2 ( Donaldson, 1994). Papadakis et al. reported similar findings in that, among board certified internists in the United States, those with lower ratings for professionalism experienced higher levels of discipline by state licensing boards ( Papadakis et al., 2008).

There is evidence suggesting that training and/or competence can help predict which physicians will be referred for fitness-for-duty evaluations. Humphrey et al., ( Humphrey, Hickman, Gulliford, 2011) examined a group of physicians in the UK and found significantly higher levels of discipline reported at all levels for physicians who trained outside of the UK as opposed to those trained in the UK ( Humphrey, Hickman, Gulliford, 2011). In this case, the authors appear to suggest that location of training is a proxy for the quality of training, and that poorer training (in this case, considered training outside the UK) resulted in more referrals for fitness-for-duty evaluations. A study by Yates examined UK medical school graduates with proven misconduct by the medical board. It was found that these physicians had experienced significantly increased incidence of academic difficulty during medical school ( Yates, James 2010).

In their study of US physicians who entered internal medicine residency training from1990 to 2000, and who went on to become board certified internists, Papadakis et al., (2008) reported that high performance on the American Board of Internal Medicine (ABIM) certification examination was associated with lower discipline by state licensing boards ( Papadakis et al., 2008). A retrospective follow up study by Lipner et al. involved physicians enrolled in accredited internal medicine residency programs within the US and showed that disciplinary actions were lowest for those achieving board certified status in internal medicine, slightly higher for those with certifications in other disciplines, and significantly higher for those who had not achieved board certification in any area of expertise ( Lipner et al., 2016). The available data from the UK and US suggests that higher levels of training may protect against referral for a fitness-for-duty evaluation.

Demographic variables may also affect fitness-for-duty evaluations. Unwin and colleagues’ examination of sanctions against physicians registered to practice in the UK found that female physicians had nearly one-third the odds of receiving sanctions as compared to males, although this finding varied by specialty ( Unwin et al., 2014). Yates’ study of UK physicians with serious medical misconduct found that men, and physicians from what were considered to be lower social classes, had a higher likelihood of misconduct ( Yates, James, 2010).

**Table 2.** Empirical results of studies examining physicians who have possible impairment in their roles as physicians.

Study	Design	Number of subjects	Type of subjects	Variables measured	Outcome	Specialty
Campbell et al., 2013	Recording of fitness-for-duty reports for anesthesiologists in the United Kingdom to the General Medical Council 2009.	8,978	Anesthesiologists in the United Kingdom.	Fitness-for-duty reports on anesthesiologists.	Reports were for clinical care and “probity.” 0.83% of practitioners received fitness-for-duty evaluations each year. This was less than other specialties.	Referrals to board by each specialty (%): Anesthesia 1; General Practice 2; Medicine 2; Non GP/Specialist 1; Obstetrics and Gynecology 4; Ophthalmology 2; Pediatrics 2; Pathology 1; Psychiatry 5; Radiology 1; Surgery 4.
Donaldson 1994	Problem doctors in a National Health Services (NHS) workforce over a five-year period.	850	Senior physicians working in the National Health Service’s northern health region.	Problems encountered by doctors in their practice serious enough to warrant possible disciplinary action.	Six percent of all senior medical staff (49/850) were referred. Problems were categorized as 1) poor attitudeand disruptive or irresponsible behavior (32); 2) lack of commitment to duties (21); 3) poor skills and inadequate knowledge (19); 4) dishonesty (11); 5) sexualmatters (7); 6) disorganized practice and poorcommunication with colleagues (5); and 7) other problems (1). Twenty-five of the 49 doctors retired or left the employer’s service, whereas 21 remained in employment after counselling or under supervision.	Doctor’sSpecialty (n=49)Anesthesiology 7; General Medicine 2;General Surgery 4;Geriatrics 4;Obstetrics and Gynecology 5;Other Medical (including Pediatrics) 6Other Surgical 9; Psychiatry 11; Radiology 1.
Finlayson et al., 2013	Retrospective comparison of results of fitness-for-duty evaluations of those referred for disruptive behavior compared to other groups.	381	Physicians referred to the Vanderbilt Assessment Service.	Psychiatric assessment to determine fitness to practice.	36.7% had one Axis I diagnosis, 20.5% had two, 10.5% had three, and 1.8% had four. The presence of an Axis I diagnosis reduced the chances of being found fit-for-duty. Those presenting with disruptive behavior were statistically significantly less likely to be assessed as unfit for practice compared to substance use, mental health, and sexual boundary groups. Specialties that had the highest percentage in the evaluation group compared to their presence in the physician population were Family Medicine (10.9/16.3); Obstetrics (4.7/9.2) and Surgery (13.7/20.5).	The largest represented specialties were Internal Medicine 41.4%; Surgery 13.7%; Family Medicine 10.9% and Pediatrics 8.7%.
Humphrey et al., 2011	A retrospective cohort study of physicians disciplined in the United Kingdom.	6,954	Physicians practicing in the United Kingdom.	Probability of discipline at various levels of discipline.	There were significantly higher levels of discipline at all levels for physicians who trained outside the United Kingdom.	
Papadakis et al., 2008	Retrospective.	66,171	US Physicians who entered internal medicine residencytraining 1990 to 2000 and becamediplomates.	Discipline action by state licensing boards.	A low professionalism rating as a residentassociated with a high risk for discipline.A high performance onthe ABIM certification examination was associated with lower discipline.	
Unwin et al., 2014	Retrospective study.	329,542	Physicians registered to practice medicine in the UK in 2013.	Sanctions on a doctor’s medical registration.	Females had nearly one-third the odds of having sanctions compared to males. This finding varied by specialty.	
Yates, 2010	Retrospective case study.	59 cases;236 controls	UK medical school graduates with serious professional misconduct and controls.	Proven misconduct by the General Medical Council.	Cases were more likely to be men, tobe of lower social class, and to have hadacademic difficulties during their medical course.	
Lipner et al., 2016	Retrospective follow-up study.	66,881	Physicians enrolled in accredited internal medicine residency programs.	Medical board disciplinary actions.	Disciplinary actions were lowest for those achieving board certified status in internal medicine, slightly higher for those with certifications in other disciplines, and significantly higher for those who never achieved any board certification.	

*Findings from the literature on how to perform fitness-for-duty evaluations.* Fitness-for-duty evaluations are, at their core, psychiatric evaluations. As such, these evaluations should meet the standards of a high-quality psychiatric evaluation along with collection of a thorough history, collateral information, and job performance data. In addition, if indicated, psychometric and laboratory testing should be performed.

In a patient psychiatric evaluation, the interests of the evaluator and the client are relatively in sync. That is, both doctor and patient are focused on treatment and improvement in functioning. However, a fitness-for-duty evaluation is fundamentally different from evaluations performed by treatment providers. It is important that the professional under evaluation is made aware of the nature of the interview, the associated limits of confidentiality, and the fact that the evaluator is not acting in the role of a treatment provider. Any issues of potential bias on the part of the evaluator should be disclosed in advance. It is also advisable to clarify the questions in writing with the person who referred the physician for treatment.

In general, fitness-for-duty reports should provide an opinion on the presence of an emotional disorder and whether the disorder interferes with the physician’s job performance. Final decisions as to whether a physician can perform his or her duties will ultimately be decided by physicians with a similar professional background. Reports should include pertinent positives and negatives relevant to the evaluator’s opinion; however, evaluators should give some consideration as to who will have access to the report, and keep confidentiality concerns in mind when deciding on the level of detail to include regarding case-specific issues. As is the practice when completing forensic reports, care should be taken to ensure that confidential health information is not inappropriately distributed.

## Discussion

The literature reviewed indicates that small but significant numbers of physicians will be referred for fitness-for-duty evaluations and, of these, perhaps one percent with relatively serious indications. We know that prior performance of some physicians, dating back to medical school, can serve as an indicator of future unprofessional conduct. The key factors associated with deficiencies in professionalism relate to personality (e.g. an inability to learn from mistakes and/or a poor display of responsibility, initiative, and motivation.) and culture (professionalism). As we move from medical student to full-fledged physician, factors such as quality of training, emotional health, and choice of subspecialty are associated with varying levels of risk. It is our hope that this review may lead to improvements in the identification and treatment of physicians whose struggles are negatively impacting their professionalism and the level of care provided to their patients.

This brings us back to our two vignettes, which were based on the authors’ experiences when working with colleagues in need of a fitness-for-duty evaluation. The first vignette touched on a number of risk factors: psychiatric specialty; personality traits (e.g., over-sensitivity and reactivity); and somewhat marginal training (e.g., never became board certified). This example showed how some limitations can be overcome with support. The doctor in this vignette never had difficulties prior to the death of his colleague and close confidant. In this case, some guidance from the medical board was all that was needed to bring the doctor’s practice up to the appropriate standard of care. Of note, despite the presence of some risk factors that tend to be less amenable to change (e.g., personality), the physician was able to respond at a level that allowed him to practice medicine independently once again.

In our second vignette, the protective effects of solid training and aptitude kept this physician from displaying obvious impairments, until the point when his life essentially fell apart. As in the first vignette, this physician also experienced a reduction in level of social support. However, the concomitant development of a significant mental health syndrome, compounded by his marital difficulties, eventually led to the physician behaving in ways that jeopardized his career. Fortunately, these factors were remediable with support, and he was able to return to successful practice.

Overall, a well performed fitness-for-duty evaluation can be beneficial to the doctor, his organization, and the public.

## Take Home Messages

• Fitness-for-duty referrals are common – at least one percent a year for physicians.
• Risk factors for fitness-for-duty evaluations include training factors, personality factors, cultural factors and emotional disorders.
• Many risk factors can be modified or improved with training or modifications. These include deficiencies in training, cultural education and treatable emotional disorders.
• Most physicians with fitness for duty issues eventually return successfully to practice.

## Notes On Contributors

James Reich, MD is Clinical Professor of Psychiatry at UCSF Medical Center and Consulting Porfessor of Psychiatry at Stanford Medical School. He is the founder of the Association for Research in Personality Disorders (ARPD) and has published in the areas of anxiety, personality and forensic medicine.

Michael Kelly, MD is a board certified forensic psychiatrist who is a Clinical Instructor at Stanford Medical School Department of Psychiatry. He is also a staff physician at the San Quentin Correctional Facility.
