# Supplementary material for: Empirical findings of fitness-for-duty evaluations
Source: MedEdPublish (2016). 2018 Nov 14;7:258. [Version 1] doi: 10.15694/mep.2018.0000258.1 (PMC10711949; doi:10.15694/mep.2018.0000258.1)
Supplement: Peer review history

# Reviewer report: Reviewer response for version 1

**DOI:** 10.21956/mep.19683.r29407

**Published:** 2019-06-08

**Authors:** Ken Masters

**Affiliations:** 1 Sultan Qaboos University

This review has been migrated. The reviewer awarded 3 stars out of 5 The paper is a literature review of empirical findings of fitness-for-duty evaluations of physicians.Some issues with the paper:• The title should reflect that it is a literature review (I realise that the authors have selected that category, but, when papers are listed in other sites, such as indexing sites, the category is not listed, so, having it in the title is recommended).• The Introduction is far too short, and gives very little sense of the scope of the problem that is being addressed.• The Methods section is also far too short. While this is not billed as a systematic review, far too few details about the search (no dates of search, search phrase(s), Boolean operators, main terms, filtering, etc) are given.• While the Vignettes make for interesting reading, it is not clear what they add to the paper as a review of the literature. The paper does return to them in the Discussion, but really does not add to the value of the review. The paper really should confine itself to reviewing the literature only.• While the Results are valuable, again, the presentation interferes with the message. This section should begin with the number of studies found, then Table 1 and Table 2, and should refer to the Tables in the text. Then follow with the description. (If the authors wish to leave the Tables for later in the Results, that is fine, but then it is essential that they refer to them in the text).So, from the Results, it appears that the authors have done the hard work correctly, but have not documented it well, and have not presented the paper in a way that justifies their effort.

Reviewer Expertise:

NA

No decision status is available

# Reviewer report: Reviewer response for version 1

**DOI:** 10.21956/mep.19683.r29408

**Published:** 2019-03-15

**Authors:** Richard Hays

**Affiliations:** 1 James Cook University

This review has been migrated. The reviewer awarded 4 stars out of 5 I found this paper both interesting and sobering. The topic is so important that I am surprised that it has drawn little comment. The review appears comprehensive and provides evidence that confirms most of the professions’ views and fears. I have some tips for possible improvement. First, the context appears to be US- focused, although it is not really when the literature is reported and analysed. It may be more appealing internationally if terms were explained. For example, is “fit-for-duty” the same as “fitness for practice”? Second, the use of the vignettes, which appear sound, amidst a literature review is potentially confusing. Did they guide (even constrain) the literature search, or ar they designed as educational strategies based on the review? I think it is the latter, but their location before the results are presented makes this less clear. Third, why was only PubMed searched? There are other sources. Fourth, how did the clinical background of the author (psychiatry) influence the review? It is possible that there was a focus on mental health when there may be other causes. Finally, readers would benefit from some discussion on implications for teaching practice, based on the review. Overall I think this is a useful contribution that might guide readers to summaries of available evidence.

Reviewer Expertise:

NA

No decision status is available
